# Impact of digital economy on residents’ medical cost: an empirical analysis based on interprovincial data from China

**DOI:** 10.3389/fpubh.2025.1742511

**Published:** 2026-01-21

**Authors:** Kai Zheng, Yan Guo, Rui Zhang, Jianjun Wu, Aiping Ma, Shenwen He, Yiwei Huang, Heng Wang, Sheng Lin, Yongyue Li, Xuejun Wang

**Affiliations:** 1 School of Public Health, Gansu University of Chinese Medicine, Lanzhou, Gansu, China; 2 School of Healthy management, Gansu University of Chinese Medicine, Lanzhou, Gansu, China

**Keywords:** digital economy, impact effect, mechanism of action, medical expenditure, provincial panel data

## Abstract

**Introduction:**

This study aims to investigate the impact of the digital economy on residents’ medical expenditure, and the underlying causes.

**Methods:**

Utilizing panel data from 30 provinces in China spanning 2017 to 2021, we employed time-fixed effects models, mediation models, and panel threshold models to analyze the influence of the digital economy on residents’ medical expenditure.

**Results:**

Research findings showed that the well-developed digital economy in a certain region significantly reduces local healthcare expenditure, while substantially increased it for the neighboring regions. This impact varied across different areas and is influenced by the regional age structure. The Consumer Price Index (CPI) exhibited a masking effect that weakened the direct impact of digital economy level on healthcare expenditure.

**Discussion:**

It is recommended to accelerate information technology infrastructure construction, enhance coordination among healthcare, pharmaceutical, and medical insurance policies, and promote technological innovation and product development that better suit the older adults.

## 1 Introduction

Health stands as a fundamental pursuit for all humanity, with profound implications for an individual’s quality of life and economic productivity, irrespective of a country’s development status. However, the rapid growth of healthcare costs, both in China and globally, has placed a significant economic burden on patients and society at large, underscoring the urgency for effective cost-containment strategies. Against this backdrop, the digital economy, characterized by data as a key production factor and driven by information networks and digital technology integration, offers new avenues for addressing long-standing challenges within the traditional healthcare sector.

In China, the national strategy explicitly promotes the deep integration of the digital and real economies. The “Overall Plan for Building a Digital China” (February 2023) and the “Action Plan for the Construction of Digital China in 2025” (May 2025) have outlined key tasks, including advancing institutional innovation and “AI+” initiatives, providing strategic guidance for digital healthcare applications. Chinese scholarly research has primarily focused on how digital technology integration drives the transformation and upgrading of the healthcare industry. Li and Guo (1) conceptualized new medical service models built on physical facilities, centered on big data, and delivered via high-speed networks, forming integrated online-offline systems. Li (2) highlighted that information technology not only enhances healthcare efficiency but also creates new opportunities for primary institutions. Furthermore, Chen et al. (3) emphasized the necessity of improving digital infrastructure and strengthening regional coordination to achieve deep integration between the digital economy and medical services, thereby fostering systemic quality improvements. Collectively, these studies establish a domestic research framework centered on “technology empowerment, system optimization, and quality enhancement.”

Internationally, scholars have placed greater emphasis on institutional innovation and multi-stakeholder collaboration in the digital transformation of healthcare, alongside specific technological applications. For instance, Levin-Zamir and Bertschi (4) demonstrated that collaborative planning among governments, businesses, and social organizations can enhance service capabilities and bridge the digital health gap. Stoumpos and Kitsios (5) argued that establishing operational mechanisms for digital health ecosystems should be a future priority, requiring tailored strategies for different stakeholders. At the technological level, studies in various countries have shown the tangible benefits of digital tools. Haleem and Javaid (6) found that digital technologies significantly improve long-term doctor-patient interaction quality and bring operational benefits to institutions, akin to applications like telemedicine and AI-assisted diagnostics prevalent in developed nations. Guan et al. (7) further stressed the crucial role of big data and AI in enabling intelligent and personalized healthcare services, reflecting a global trend also observed in initiatives within developing countries seeking to leapfrog traditional infrastructure limitations.

### 1.1 Comparative context and research gap

While existing literature provides valuable insights, a significant research gap remains. Most studies focus on specific technological applications or institutional reforms without systematically analyzing the overall impact of the digital economy on residents’ total healthcare expenditures. This is particularly relevant in China, where regional disparities and unique institutional backgrounds—such as varying levels of digital infrastructure and regional healthcare policies—necessitate a province-level analysis. Moreover, while international research from the EU, U. S., and Japan highlights the role of multi-stakeholder collaboration and advanced digital tools in controlling costs, these findings may not fully apply to China’s context due to differences in healthcare systems and digital adoption patterns. For example, the EU’s cross-border health data infrastructure or the U. S.’s emphasis on private-sector-led digital health innovations contrast with China’s state-driven, regionally coordinated approach. This gap underscores the need for a comprehensive study that integrates regional characteristics and institutional contexts to explore the dual effects of the digital economy—both reducing and potentially increasing costs through mechanisms like price transmission or regional resource imbalances.

Driven by growing health awareness and demand for efficient, convenient, and personalized services, investments in digital medical technology and platforms by Chinese hospitals and governments have fueled the expansion of the digital health market (8). The digital economy has increased the transparency and controllability of personal healthcare expenditures (9), allowing patients to compare prices and service quality across institutions and medications via online platforms, leading to more informed decision-making (10). Conversely, the widespread adoption of digital technologies also carries risks, such as over-reliance on advanced medical equipment for diagnostics or excessive promotion of digital services, potentially increasing residents’ medical expenditures (11). Existing research often focuses on the cost implications of digital technologies in specific healthcare processes, failing to capture the overall changes in residents’ total healthcare expenditures and the underlying mechanisms. Therefore, this study aims to comprehensively investigate how the digital economy affects residents’ medical costs from multiple perspectives. This will assist the government in better understanding the mechanisms through which the digital economy influences medical expenditures and in formulating more precise and effective healthcare policies.

### 1.2 Structure of the paper

The remainder of this paper is organized as follows: Section 2 outlines the theoretical mechanisms and research hypotheses, including pathways of technological empowerment, spatial spillover effects, and the roles of CPI and population aging. Section 3 describes the methodology, data sources, and variable construction. Section 4 presents the empirical results, including spatial correlation analysis, regression outcomes, robustness checks, and heterogeneity analysis. Section 5 discusses the findings in relation to existing literature and policy implications. Section 6 concludes with recommendations for leveraging the digital economy to optimize healthcare expenditure governance in China.

## 2 Mechanism analysis and research hypothesis

Based on theoretical analysis and policy context, this paper constructs an integrated analytical framework to elucidate the logical mechanism through which the digital economy affects residents’ healthcare expenditure. The framework posits “Technological Empowerment” as the foundational driver, which facilitates “Institutional Change” in healthcare payment systems and induces “Price Transmission” via the Consumer Price Index. Furthermore, the framework recognizes that the effectiveness of these pathways is moderated by the regional “Population Structure,” specifically the aging level. This structured approach allows us to examine both the direct effects and the interplay among these dimensions.

### 2.1 The path of service efficiency improvement empowered by digital technology

The digital economy promotes the evolution of medical service models towards online, intelligent, and collaborative directions by optimizing the information flow and resource flow in the medical service system. Specifically, by relying on electronic health records, teleconsultation platforms, and intelligent appointment management systems, digital medical information platforms have achieved interconnectivity of data across institutions, effectively reducing inefficient medical expenditure such as duplicate examinations and redundant treatments. Furthermore, the digital transformation of medical treatment processes has enhanced service efficiency while reducing the time and financial costs incurred by patients during registration, payment, and waiting periods. These reforms collectively contribute to the reduction of individual healthcare expenditure among residents. On the other hand, with the advancement of digital economy, the medical insurance payment system is also gradually reformed into a diagnosis-related group (DRG) or diagnosis-intervention packet (DIP) based payment mechanism for specific diseases. Furthermore, the advancement of the digital economy provides the technological infrastructure necessary for implementing sophisticated healthcare payment reforms, such as Diagnosis-Related Groups (DRG) or Diagnosis-Intervention Packet (DIP) systems. Digital platforms enable real-time aggregation and standardization of healthcare cost and outcome data, which provides an essential data foundation for medical insurance authorities to accurately calculate disease grouping (DRG) and payment standards. This, in turn, lowers the implementation barriers and information costs of institutional reform and accelerates the rollout of payment method reforms. While these reforms are primarily policy-driven initiatives aimed at cost control and efficiency enhancement (12), digital platforms are indispensable for their operation, enabling precise service pricing, cost review, and medical insurance expenditure control. By preventing overmedicalization and resource wastage, the synergy between digital tools and payment reform policies can further alleviate residents’ medical expenditures (13, 14). Thus, the digital economy acts as an enabler for institutional changes that impact costs.

Based on the above analysis, this paper proposes Hypothesis 1.

*H1*: The digital economy can significantly reduce residents’ medical expenditure.

### 2.2 Spatial spillover effects and regional heterogeneity

The expansion of the digital economy has significant spatial correlation characteristics. On the one hand, the construction of digital infrastructure and the promotion of platform technologies have spillover effects, which can drive service collaboration and resource sharing in surrounding areas, thereby forming a linkage effect of “technological spillover—resource optimization—cost reduction” among neighboring regions (15, 16). For example, under the background of cross-regional recognition of medical information platforms, residents in marginal areas can indirectly enjoy high-quality services by leveraging the medical platform resources of developed areas, thereby reducing the costs of seeking medical treatment across regions.

However, the infrastructure and human resources required for the digital economy exhibit strong regional agglomeration characteristics. Developed regions have higher levels of informatization, sufficient financial investment, and high-quality medical institutions, which enable them to apply digital medical technologies more quickly and comprehensively, thereby releasing the benefits of reducing expenditure. In contrast, remote or less developed regions, due to their weak digital infrastructure and shortage of professional talents, find it difficult to effectively implement digital medical services. As a result, medical services in these areas still rely on traditional models and are unable to fully enjoy the cost-reducing effects of the digital economy (17, 18). This regional disparity implies that the impact of the digital economy on medical expenditure may not be entirely endogenous, but rather has significant spatial spillover characteristics. Based on this, this paper proposes Hypothesis 2.

*H2*: The digital economy has significant spatial spillover effects on residents’ medical expenditure.

### 2.3 The mediating transmission path of the Consumer Price Index

In the process of deep integration between the digital economy and traditional industries, enterprises need to invest a large amount of funds in infrastructure upgrades, system construction, and technical training to complete digital transformation, thereby increasing costs such as labor and raw materials (19). To maintain profits, companies often pass these costs on to consumers by raising the prices of products and services, which in turn drives up the overall Consumer Price Index (CPI). As medical services and pharmaceuticals constitute rigid consumption categories for residents, their price formation is not an isolated process but is closely linked to the general price level. On the one hand, a rise in the general consumer price index (CPI) will lead to a universal increase in the prices of production factors; as a result, the labor, consumable, and operational costs involved in the provision of medical services, as well as the raw material, R&D, and distribution costs required for pharmaceutical production, will all rise in tandem with the overall price level. On the other hand, the digital upgrading of medical services driven by the digital economy (such as the construction of telemedicine platforms and investment in intelligent diagnosis and treatment equipment) will also generate special cost increments in the healthcare sector. Combined with the transmission effect of the general CPI, these factors jointly drive up the prices of medical services and pharmaceuticals. The rigid demand nature of medical services and pharmaceuticals results in their low price elasticity: residents’ demand for medical services will not decrease significantly with price hikes. Therefore, the increase in the prices of medical services and pharmaceuticals will be directly translated into a rise in residents’ healthcare expenditure. In addition, consumer preferences have also undergone structural changes in the digital economy environment, evolving from basic and functional consumption to more personalized, high-quality, and high-frequency consumption (20), which further raises the overall level of expenditure.

The rise in CPI means that residents have to bear higher expenditure on goods and services in addition to the basic cost of living, which may partially offset the positive effects of the digital economy on the efficiency and transparency of medical services. The change in consumer prices may act as a mediating variable in this mechanism. Therefore, this paper proposes Hypothesis 3.

*H3*: The Consumer Price Index partially mediates the impact of the digital economy on residents' medical expenditure.

### 2.4 The moderating effect path of population aging

With the acceleration of the aging process in China, the health needs of the older adults are growing rapidly. Evidence shows that aging is associated with increased prevalence of chronic conditions and greater demand for long-term care services, making chronic disease management, long-term care, and rehabilitation the main areas of expenditure (21, 22). This demographic shift creates health expenditure patterns characterized by inherent rigidity that are less responsive to changes in service delivery models (11).

Concurrently, older adults generally exhibit significant challenges in adapting to digital technologies. Research demonstrates that older adults face substantial information barriers and display technological aversion when confronted with complex online service systems (23). Studies specifically document a low prevalence of adequate eHealth literacy and willingness to use telemedicine among older adults, which hampers their effective utilization of digital health resources. This limited digital adoption is particularly problematic in healthcare contexts where technology acceptance is crucial for service accessibility.

The combination of these factors creates a unique challenge for digital economy interventions in aging societies. On one hand, the structural nature of older adults’ healthcare needs creates expenditure patterns that are inherently resistant to modification through efficiency improvements alone. On the other hand, the low utilization rate of digital services among older populations fundamentally weakens the implementation of cost-reducing effects that digital economy solutions might otherwise provide. This is particularly evident in contexts where digital healthcare transformation requires significant behavioral adaptation from users (5).

Therefore, when regional aging levels surpass a certain threshold, the mitigating effect of the digital economy on residents’ medical expenditure is likely to be significantly weakened. Based on this reasoning, the following hypothesis is proposed:

*H4*: The mitigating effect of the digital economy on residents' medical expenditure will no longer be significant when the level of aging reaches a certain threshold.

## 3 Methods and data

### 3.1 Spatial autocorrelation analysis

To examine the spatial autocorrelation of the digital economy and residents’ medical expenditure, this study employed both global and local Moran’s I indices. The calculation of these indices relies on a standardized spatial weights matrix, expressed as follows (Equation 1):


$$I=\frac{\sum_{i=1}^{n}\sum_{j=1}^{n}w_{\mathrm{ij}}(x_{i}-\bar{x})(x_{j}-\bar{x})}{\sum_{i=1}^{n}{\left(x_{i}-\bar{x}\right)}^{2}}$$ (1)

In the formula, $x_{i}$ represents the observed value of region i , nrepresents the total number of regions, and $w_{\mathrm{ij}}$ represents an element of the spatial weights matrix, which is used to define the proximity relationships between different spatial units. This study primarily employs a spatial contiguity weights matrix. It is defined such that if two regions share a common border or vertex, they are considered contiguous and assigned a weight of 1; otherwise, the weight is 0. The mathematical expression is as follows (Equation 2):


$$W_{\mathrm{ij}}(D)=\{\begin{array}{c} 1,d_{\mathrm{ij}}\leq D\\ 0,d_{\mathrm{ij}}>D \end{array}$$ (2)

Where $d_{\mathrm{ij}}$ represents the geographical distance between the administrative centers of region i and region j , and D is a specified distance threshold. It is important to clarify that in the empirical setting of this paper, the distance threshold D is defined as an extremely small value. This ensures that the condition $d_{\mathrm{ij}}\leq D$ is met only when two regions are actually adjacent, thereby constructing a contiguity matrix based on shared borders. This type of weights matrix is a standard method in spatial econometrics for characterizing geographical contiguity.

This contiguity-based matrix is chosen as the primary spatial weights matrix because it intuitively captures the concept of “geographical contagion” or immediate neighbor effects, which is theoretically relevant for the diffusion of technological innovations and healthcare-seeking behaviors across provincial borders (24, 31).

### 3.2 Model construction

#### 3.2.1 Benchmark regression mode

When analyzing the medical expenditure of residents, to mitigate the endogeneity issue caused by missing variables, as well as the time trend or exogenous shock of the COVID-19 pandemic that has the same impact on all regions across the country, eliminate its interference with the medical expenditure of residents, and focus the model more on individual differences and the impact of the digital economy by using time fixed effects to construct the basic model as follows (Equation 3):


$$\mathrm{OO}P_{\mathrm{it}}=\alpha +\beta_{0}\mathrm{DE}I_{\mathrm{it}}+\beta_{1}LS_{\mathrm{it}}+\beta_{2}GI_{\mathrm{it}}+\beta_{3}HR_{\mathrm{it}}+\beta_{4}EH_{\mathrm{it}}+\gamma_{t}+\varepsilon_{\mathrm{it}}$$ (3)

Where OOP_it_ represents the medical expenditure of residents in province i in year t, and DEI_it_ represents the level of the digital economy in province i in year t, LS_it_ represents the living standard of residents in province i in year t, and GI_it_ represents the level of government intervention in province i in year t, HR_it_ represents the level of health resources in province i in year t, EH_it_ represents the livability of the environment in province i in year t, *α* is a constant, λt represents the time fixed effects, and ϵ_it_ is the random disturbance term.

#### 3.2.2 Mediating effect model

To examine the influence pathway between the development of the digital economy and the medical expenditure of residents, this study employs a stepwise regression method to test the mediating effect based on Equation 3. The specific steps are as follows:

First, analyze the total effect of the digital economy on the medical expenditure of residents, as shown in the following (Equation 4).


$${\mathrm{OOP}}_{\mathrm{it}}=\alpha +\alpha_{0}{\mathrm{DEI}}_{\mathrm{it}}+\alpha_{1}X_{\mathrm{it}}+\gamma_{t}+\varepsilon_{\mathrm{it}}$$ (4)

Second, examine the impact of the digital economy on the mediating variable, where CPI_it_ represents the Consumer Price Index (CPI) of residents, as shown in the following (Equation 5).


$${\mathrm{CPI}}_{\mathrm{it}}=\beta +\beta_{0}{\mathrm{DEI}}_{\mathrm{it}}+\beta_{1}X_{\mathrm{it}}+\gamma_{t}+\varepsilon_{\mathrm{it}}$$ (5)

Third, examine the direct and indirect effects of the digital economy on the medical expenditure of residents through the mediating variable, as shown in the following (Equation 6).


$$\mathrm{OO}P_{\mathrm{it}}=\gamma +\gamma_{0}\mathrm{DE}I_{\mathrm{it}}+\gamma_{1}\mathrm{CP}I_{\mathrm{it}}+\gamma_{2}X_{\mathrm{it}}+\gamma_{t}+\varepsilon_{\mathrm{it}}$$ (6)

Where control X_it_ represents the control variables.

#### 3.2.3 Spatial econometric model

In regional panel data, spatial correlation often leads to biased estimates in OLS or traditional fixed-effects models (24). To identify and correct for potential spatial spillover effects in the impact of the digital economy on the medical expenditure of residents, this study introduces a spatial econometric model for further analysis. Spatial econometric models mainly include three basic types: Spatial Error Model (SEM), Spatial Autoregressive Model (SAR), and Spatial Durbin Model (SDM).

Among them, the SEM is used to correct the estimation bias caused by spatial correlation in the error term, the SAR is used to capture the spatial spillover effects of the dependent variable, while the SDM takes into account both the spatial lag of the dependent variable and the explanatory variables, making it a more general extended model that can simultaneously control the impact of neighboring regional characteristic variables.

To determine the most suitable model specification for this study, model selection analysis is conducted based on the LM (Lagrange Multiplier) test, LR (Likelihood Ratio) test, Wald test, and Hausman test.

The specific results are shown in Table 1. At the 5% significance level, the LM test significantly rejects the null hypothesis, indicating the presence of spatial effects in the sample; the LR and Wald test results support that the SDM outperforms the SAR and SEM; the Hausman test indicates that the fixed-effects model is superior to the random-effects model; the results of the LR test show that the individual differences across provinces have no statistically significant impact on the model, while the time dimension contributes significantly to it. This indicates that the time dimension must be included in the model, whereas the individual dimension can be considered for exclusion. Therefore, in the subsequent empirical analysis, this study adopts the Spatial Durbin Fixed-Effects Model (SDM-FE) as the main estimation framework to more comprehensively evaluate the spatial diffusion effect of the digital economy and its impact on the medical expenditure of residents.

**Table 1:** Relevant tests of spatial econometric models.

Inspection type	Spatial contiguity weights matrix	
	Value	*p*-value
Moran’s I	1.829	0.067
LM-error	2.476	0.116
LM-lag	6.835	0.009
LR-SDM/SAR	18.72	0.000
LR-both/time	275.56	0.000
LR-both/ind	6.61	0.158
Wald- SDM/SEM	38.28	0.000
Wald- SDM/SAR	56.97	0.000
Hausman	38.45	0.000

The formula for the Spatial Durbin Model used in this study is as follows (Equation 7):


$$\mathrm{OO}P_{\mathrm{it}}=\gamma_{0}+\text{ρWOO}P_{\mathrm{it}}+\gamma_{1}\mathrm{DE}I_{\mathrm{it}}+\theta_{1}\mathrm{DE}I_{\mathrm{it}}+\gamma_{i}X_{\mathrm{it}}+\theta_{i}X_{\mathrm{it}}+\gamma_{t}+\varepsilon_{\mathrm{it}}\;$$ (7)

Where *ρ* represents the spatial autoregressive coefficient, W represents the spatial weights matrix, $\theta_{1}$ and $\theta_{i}$ represent the spatial effect coefficients, X_it_ represents the control variables, including government intervention, health resources, environmental livability, and the living standard of residents, and ϵ_it_ is the random error term. The model is capable of capturing the spatial spillover effects of both the dependent variable and the explanatory variables simultaneously, making it more suitable for analyzing the spatial transmission pathways of digital economic development on medical expenditure across regions.

### 3.3 Variable definitions and data sources

#### 3.3.1 Dependent variable: medical expenditure of residents

This study primarily uses the proportion of personal health expenditure in total health expenditure as the core indicator to measure the medical expenditure burden of residents. Personal health expenditure refers to the actual cash payments made by urban and rural residents when receiving various medical and health services. This includes out-of-pocket payments required under various medical insurance schemes. This indicator accurately reflects the relative economic burden borne by individuals in overall health expenditure and is highly representative for assessing the intensity of residents’ medical expenditure.

#### 3.3.2 Core explanatory variable: digital economy index

When assessing the development of the digital economy, domestic scholars typically consider various dimensions of the digital economy, such as digital infrastructure, digital industries, and output efficiency.

This study draws on the approach of Liu et al. (25).who use internet development as the core measure and incorporate indicators of digital transactions. This study measures the level of digital economic development in various regions of China from two aspects: internet development and digital financial inclusion, considering the availability of relevant data, as shown in Table 2.

**Table 2:** Indicator system for the level of digital economy.

Primary indexes	Secondary indexes	Indexes attribute
Internet development	Fiber optic cable density	+
	Number of mobile phone base stations	+
	Internet access port density	+
	Mobile phone penetration	+
	Internet users per 100 people	+
	Number of employees	+
	Total telecommunications services per capita	+
Digital inclusive financial index	Breadth of coverage of the digital economy	+
	Depth of use of the digital economy	+
	Degree of digitalization	+

Specifically, the construction of the index is carried out from the following dimensions: First, internet development, from which the following indicators were selected: optical cable density, number of mobile phone base stations, internet access port density, mobile phone penetration rate, number of internet users per 100 people, number of employed persons, and per capita telecommunications business volume. All the basic data are sourced from the China Statistical Yearbook. Second, digital financial inclusion, using the China Digital Inclusive Finance Index jointly compiled by the Digital Finance Research Center of Peking University and Ant Financial Group to comprehensively reflect the digital financial development of each province, including three dimensions: the breadth of digital economy coverage, the depth of digital economy usage, and the degree of digitalization. The above 10 indicators are all positive indicators. Compared with the subjective weighting method, the entropy method eliminates human interference factors, enables an objective evaluation of the relative importance of each indicator, and has been widely applied in objective weighting within the socio-economic field. To comprehensively evaluate and ensure comparability among indicators, this paper adopts the following steps to construct the digital economy index:

First, the original data is subjected to min-max standardization to eliminate the influence of different units of measurement (Equation 8):


$$X_{\mathrm{ij}}^{\prime }=\frac{\left(X_{j}-X_{\min}\right)}{\left(X_{\max}-X_{\min}\right)},1\leq i\leq m,1\leq j\leq n\;\;$$ (8)

Next, the proportion of indicator j for province i is calculated (Equation 9):


$$P_{\mathrm{ij}}=\frac{X_{\mathrm{ij}}^{\prime }}{\sum_{i=1}^{m}X_{\mathrm{ij}}^{\prime }},0\leq P_{\mathrm{ij}}\leq 1\;\;\;$$ (9)

Then, the entropy value of indicator j is further calculated to obtain the information entropy redundancy (Equations 10, 11):


$$e_{j}=-\frac{1}{\mathrm{lnm}}\sum_{i=1}^{m}P_{\mathrm{ij}}\ln P_{\mathrm{ij}}\;\;$$ (10)

$$d_{j}=1-e_{j}\;\;\;$$ (11)

Subsequently, the weight of indicator j is calculated (Equation 12):


$$W_{j}=\frac{d_{j}}{\sum_{i=1}^{m}d_{i}}\;\;\;\;$$ (12)

Finally, the digital economy index for each province is derived as follows (Equation 13):


$$\mathrm{DEI}=\sum_{i=1}^{m}W_{j}y_{\mathrm{ij}}\;\;$$ (13)

While the DEI aims for comprehensiveness, we acknowledge that indicators like “number of employed persons” in the internet sector could be endogenous, as regions with higher economic development might have both a larger digital workforce and different healthcare spending patterns. To address this and other potential sources of endogeneity, an instrumental variable approach is employed in the robustness checks.

#### 3.3.3 Control variables

To control for potential confounding factors that are theoretically and empirically linked to residents’ medical expenditure, this study incorporates four categories of control variables, drawing on existing literature (26, 27). The selection is based on their potential to influence healthcare costs through distinct channels: government intervention reflects policy intensity, health resources indicate supply capacity, environmental livability affects population health status, and living standards capture economic capacity for healthcare consumption.

To control for potential confounding factors influencing residents’ medical expenditure, this study incorporates four control variables: Government Intervention (GI), measured as the proportion of government health expenditure in total health expenditure; Health Resources (HR), proxied by the number of health technical personnel per thousand population; Environmental Livability (EH), indicated by the urban green space coverage rate (%); and Living Standard of Residents (LS), represented by the natural logarithm of per capita regional GDP. The logarithmic transformation of per capita GDP is applied to mitigate data skewness and to allow the coefficient to be interpreted as an elasticity, capturing the percentage change in medical expenditure associated with a 1% change in per capita GDP, which aligns with standard practice in economic studies. Descriptive statistics for all variables are summarized in Table 3.

**Table 3:** Statistical description of variables.

Variables	Symbol	Mean	Sd	Min	Max
Resident’s medical expenditure	OOP	27.50	4.15	13.15	35.18
Digital economy index	DEI	0.28	0.23	0.03	1.13
Living standards	LS	11.07	0.39	10.26	12.12
Government input	GI	29.74	6.64	18.66	49.21
Environmental habitability	EH	10.59	9.66	0.64	53.29
Healthy resources	HR	7.02	1.27	4.74	12.61

### 3.4 Data sources and validation

This study utilizes panel data from 30 Chinese provinces covering the years 2017 to 2021 as the research sample, considering data availability and completeness. The Tibet Autonomous Region was excluded due to data limitations. Data sources include the China Health Statistics Yearbook, the China Statistical Yearbook, the National Bureau of Statistics official website, and the Digital Finance Research Center of Peking University. To ensure the stability of the model, this study conducted tests for multicollinearity among the variables, the results of which are shown in the table. All Variance Inflation Factors (VIF) of the variables are less than 5, as shown in Table 4, indicating that the model constructed using these variables is relatively stable and reliable.

**Table 4:** Multicollinearity test results.

Variables	Symbol	VIF
Digital economy index	DEI	1.94
Government input	GI	1.58
Environmental habitability	EH	1.44
Healthy resources	HR	1.83
Living standards	LS	3.09
Mean VIF		1.98

## 4 Results

### 4.1 Spatial correlation analysis

Before formally conducting spatial econometric modeling analysis, it is necessary to examine whether there is significant spatial correlation between the research objects — the level of digital economic development and residents’ medical expenditure. The spatial autocorrelation test can provide a theoretical basis for whether to introduce spatial effects. This paper employs the Global Moran’s I and the Moran Scatterplot to conduct a spatial clustering analysis of the two types of variables.

Table 5 presents the Moran index calculation results for the level of digital economic development and residents’ medical expenditure in 30 provinces (municipalities, autonomous regions) of China from 2017 to 2021. It can be found that: the Moran index of digital economy is positive in all years and passes the test at the 1% significance level. The Moran index of residents’ medical expenditure also shows significant positive correlation in most years (except for 2020). Overall, both show strong positive spatial correlation and spatial agglomeration characteristics, indicating that there is a clear “high-high” or “low-low” adjacency effect among regions.

**Table 5:** Moran’s I index measurement results.

Year	Resident’s medical expenditure			Digital economy index		
	Moran’s I	Z value	*P*-value	Moran’s I	Z value	*P*-value
2017	0.254	2.380	0.009	0.277	2.713	0.003
2018	0.228	2.172	0.015	0.300	2.850	0.002
2019	0.216	2.117	0.017	0.274	2.710	0.003
2020	0.119	1.344	0.089	0.261	2.583	0.005
2021	0.205	2.086	0.019	0.346	3.233	0.001

**Figure 1:**
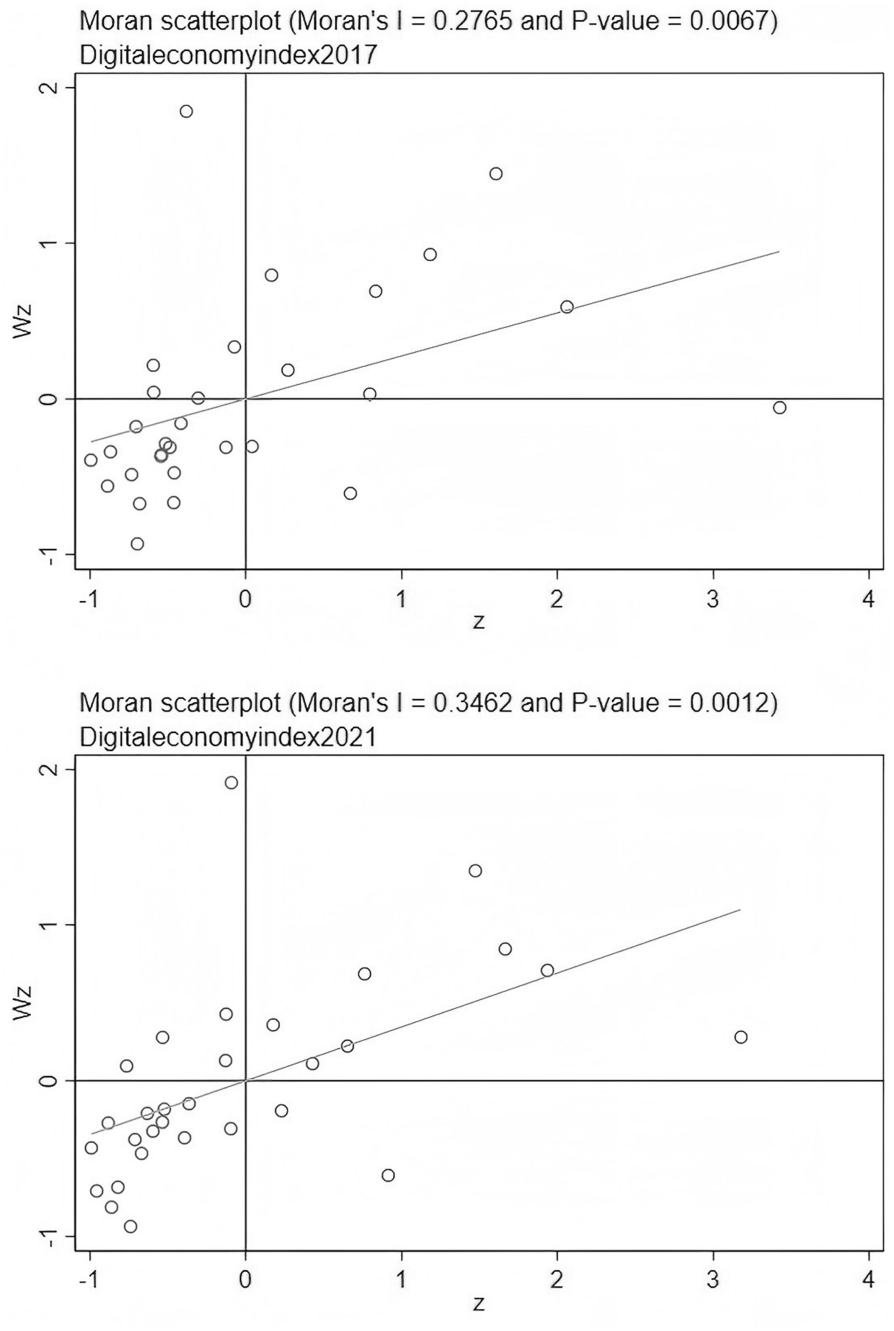
Local Moran’s I test for digital economy index.

**Figure 2:**
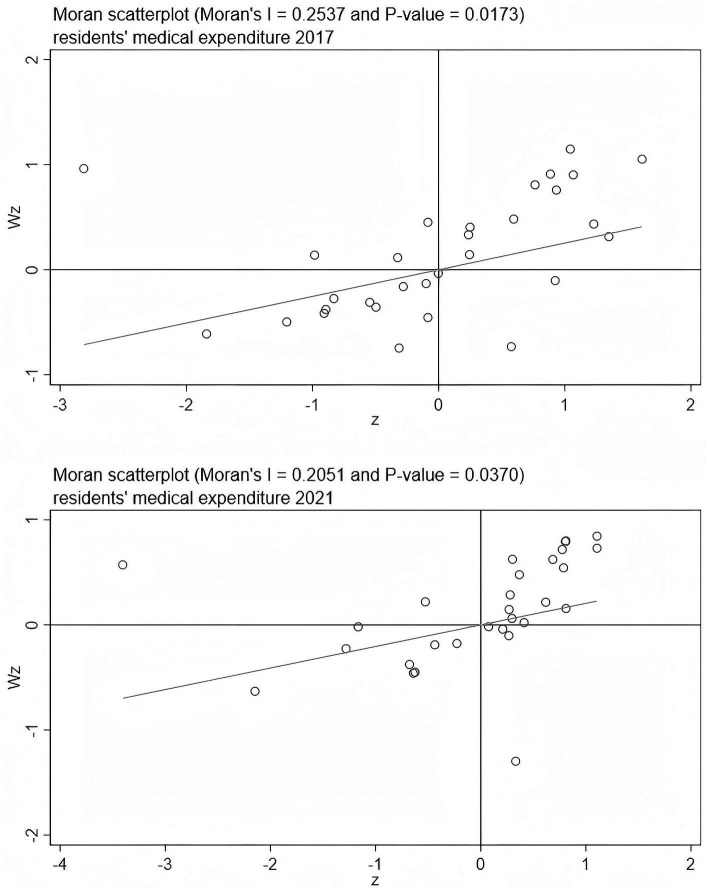
Local Moran’s I test for resident’s medical expenditure.

Furthermore, to visually display the spatial agglomeration pattern, this paper draws the Moran scatterplots for the years 2017 and 2021 (see Figures 1, 2). From the figures, it can be observed that digital economy and residents’ medical expenditure are mainly concentrated in the first quadrant (high-high) and the third quadrant (low-low) in both periods, indicating that there is strong spatial coupling between high-level or low-level regions. This spatial clustering pattern indicates that regions with similar levels of digital economic development or similar levels of medical expenditure exhibit a “convergent distribution” phenomenon in geographical space. In summary, based on the results of the Global Moran’s I and the local Moran scatterplot tests, it can be confirmed that both the digital economy and residents’ medical expenditure in China exhibit significant spatial autocorrelation and spatial agglomeration. Therefore, the introduction of spatial econometric models in the subsequent regression analysis is well-founded in both theory and empirical evidence.

### 4.2 Regression results

To accurately examine the impact of the digital economy on residents’ medical expenditure, this study initially constructed a baseline model based on Equation 3. Subsequently, based on Equation 7, the Spatial Durbin Model (SDM) was established to explore the spatial effects between the digital economy and residents’ medical expenditure. The data analysis in the research process was conducted using Stata 17.0. Table 6 presents the results of the impact of the digital economy on residents’ medical expenditure under different models. The first column of regression results shows that, without considering any control variables, the correlation coefficient between the digital economy and residents’ medical expenditure is −12.23, which is significant at the 1% level; By contrast, the regression results in the second column show that with the inclusion of control variables such as government intervention, health resources, environmental livability, and residents’ living standards, the correlation coefficient between the digital economy and residents’ medical expenditure changes to −6.99, It is significant at the 1% level, which indicates that the digital economy significantly reduces residents’ medical expenditure. The analysis of control variables shows that government intervention, health resources, environmental livability, and residents’ living standards all have a certain inhibitory effect on residents’ medical expenditure, and all are significant at the 1% level. The regression results in the third column show that after controlling for time-fixed effects, the coefficient obtained by incorporating spatial effects and control variables into the model is −6.28, which remains significant at the 1% level. This indicates that after controlling for spatial effects and time-fixed effects, the role of the digital economy in reducing residents’ medical expenditure still exhibits robustness. In addition, the coefficients of W*DEI and *ρ* are 6.79 and 0.16 respectively, both positive, indicating a significant spatial spillover effect between the digital economy and residents’ medical expenditure. The total effect and indirect effect of the digital economy on residents’ medical expenditure are positive, while the direct effect is negative. This finding confirms that although the development of the digital economy reduces the medical expenditure of residents in the local region, it increases the medical expenditure of residents in adjacent regions. From the perspective of effect decomposition, the direct effect of the digital economy is −5.977, which is significantly negative. This indicates that the development of the local digital economy effectively reduces the medical expenditure of local residents. The indirect effect is 6.965, which is significantly positive, showing that the development of the digital economy in neighboring regions, on the contrary, increases the medical expenditure of local residents; The total effect is 0.988, which is not significant, implying that the positive and negative effects basically offset each other across regions.

**Table 6:** Benchmark regressions.

Variable	(1)	(2)	(3)
DEI	−12.234*** (−10.87)	−6.993*** (−6.93)	−7.234*** (−6.15)
LS		−3.929*** (−5.14)	−2.714*** (−3.02)
GI		−0.402*** (−13.68)	−0.379*** (−12.67)
EH		−0.032*** (−3.96)	−0.057*** (−2.84)
HR		−0.666*** (−1.69)	−1.113*** (−5.20)
W*DEI			6.763*** (2.70)
_cons	30.903*** (76.69)	51.221*** (34.41)	
*ρ*			0.163* (8.61)
DEI(Direct)			−7.061*** (−5.82)
DEI(Indirect)			6.965*** (2.34)
DEI(Total)			−4.302 (−1.25)
*R* ^2^	0.440	0.787	0.740
obs	150	150	150

*, **, and *** in Tables 7–10 denote significance at the 10%, 5%, and 1% levels, respectively.

### 4.3 Robustness tests and endogeneity treatment

To test the robustness of the empirical results against different assumptions of spatial interdependence and mitigate potential endogeneity issues, this study first conducts robustness checks by redefining the spatial weight matrix.

To test the robustness of the empirical results and mitigate potential endogeneity issues, this study first conducts robustness checks by redefining the spatial weight matrix. Drawing on the methods proposed by Chen et al. (28) and Zhang et al. (29), we alternatively employ a geographic distance matrix and an inverse distance squared matrix to construct alternative Spatial Durbin Models (SDMs). The regression results, presented in Table 7, show that the coefficients for the digital economy development level (DEI) remain significantly negative (−7.55 and −8.92, respectively) at the 1% significance level under both alternative spatial weight matrices. This consistency confirms the robust impact of the digital economy on reducing residents’ out-of-pocket health expenditure.

**Table 7:** Robustness test and regional heterogeneity analysis.

Variable	Geographical distance matrix	Inverse distance matrix	Instrumental variable method	Eastern	Western	Central
DEI	−7.551*** (−6.33)	−8.922*** (−7.55)	−10.766** (−2.29)	−5.425*** (−3.51)	−4.702* (−1.52)	1.014 (0.14)
W*DEI	33.169*** (2.77)	11.573*** (2.80)		8.751*** (2.72)	17.989** (2.14)	−7.999 (−0.60)
LS	−4.189*** (−4.31)	−4.551*** (−4.85)		−6.248*** (−5.60)	−6.566*** (−4.07)	2.496 (0.87)
GI	−0.394*** (−13.39)	−0.368*** (−9.96)		−0.183*** (−2.45)	−0.071* (−1.12)	−0.505*** (−4.39)
EH	−0.137*** (−5.30)	−0.067*** (−2.94)		−0.077*** (−2.81)	0.524*** (3.35)	0.059 (0.55)
HR	−1.611*** (−6.42)	−1.434*** (−5.92)		−1.748*** (−5.97)	2.719*** (3.54)	0.248 (0.44)
DEI(Direct)	−8.737*** (−6.72)	−9.059*** (−7.04)		−5.007*** (−2.85)	−4.587** (−1.31)	2.557 (0.38)
DEI(Indirect)	24.622*** (2.72)	12.032*** (2.97)		9.134*** (2.62)	19.532* (1.80)	−7.004 (−0.66)
DEI(Total)	15.884* (1.65)	2.973* (0.67)		4.308 (0.99)	14.944 (1.10)	−4.446 (−0.29)
*R* ^2^	0.859	0.859	0.609	0.785	0.064	0.503
F-statistics			30.49			
obs	150	150	150	65	55	30

*, **, and *** in Tables 6–10 denote significance at the 10%, 5%, and 1% levels, respectively.

To address potential endogeneity bias arising from omitted variables or reverse causality, this study further employs an instrumental variable (IV) approach. The instrumental variable selected is the historical fixed long-distance telephone exchange capacity in 2003 at the provincial level. This variable satisfies the relevance condition because historical telecommunications infrastructure, as a precursor to modern digital infrastructure, is positively correlated with contemporary digital economy development (16, 25). At the same time, it plausibly satisfies the exclusion restriction. The fixed long-distance telephone exchange capacity in 2003 is a historical relic that influenced the initial conditions for digital development but is unlikely to directly affect current residents’ medical expenditure after controlling for contemporary socioeconomic factors, as these historical communication tools have been largely superseded by modern technologies (17, 29).

The two-stage least squares (2SLS) estimation results are reported in column (3) of Table 7. The coefficient for DEI is −10.76 and remains significant at the 1% level, indicating that after controlling for potential endogeneity, the digital economy continues to exhibit a significant reducing effect on out-of-pocket health expenditure. The Cragg-Donald Wald F-statistic of 30.49 comfortably exceeds the Stock-Yogo critical value, allowing us to reject the null hypothesis of a weak instrument. These tests collectively enhance the credibility of the causal interpretation of the digital economy’s impact on reducing medical expenditure.

### 4.4 Spatial heterogeneity analysis

To further examine the regional differences in the impact of the digital economy, this study, based on the national regional division standards, divides the 30 provinces into three major regions: the eastern, central, and western regions. It then constructs a spatial Durbin model within each region to examine the spatial heterogeneity characteristics. The regression results in columns four to six of Table 7 show that the impact of the digital economy on the medical expenditure of residents in the three major regions is significantly different.

Specifically, in the eastern and western regions, the total effect of the digital economy on per capita health expenditure of residents is significantly negative, and both are manifested through the direct effect as a decrease in expenditure (both at the 1% significance level). In addition, the indirect effects in the two regions are significantly positive, indicating that the development of the digital economy may lead to the agglomeration of medical resources in the region through the cross-regional flow of factors, thereby causing an increase in the medical expenditure in surrounding areas, that is, a “suction effect” exists. This result suggests that while the digital economy promotes the improvement of local health security capabilities, it may exacerbate the imbalance in the allocation of medical resources among regions.

In contrast, the digital economy variables in the central region are not significant in terms of total effect, direct effect, and indirect effect, reflecting that there may be a “digital lag” in the region in terms of digital infrastructure, resource capacity, or the level of digital application. This result also indicates that the central region may be facing the challenge of relatively slow digital economic development and limited radiation and driving capacity, thus failing to effectively alleviate the medical expenditure of residents. This may be attributed to the lack of key support for the development of the digital economy in the central regions. As traditional major provinces with large populations and the nation’s primary labor export bases, these regions have long prioritized traditional industries in their economic development and invested insufficiently in the transition to a digital economy. Meanwhile, the outflow of a large number of young and middle-aged laborers has not only weakened the demand-driven impetus for local digital medical services, but also led to a shortage in the supply of digital technology and medical professionals. This has further constrained the integrated development of digital infrastructure and medical services, ultimately resulting in a relatively limited reduction in medical expenditure.

The results reveal the characteristics of regional heterogeneity in the impact of the digital economy on residents’ healthcare expenditure. By decomposing the total effect, direct effect, and indirect effect, this study accurately identifies the spatial mechanism of action of the digital economy, demonstrating the advantages of spatial econometric methods in analyzing the regional differences in healthcare expenditure. Moreover, the results uncover the dual effects of the digital economy on healthcare expenditure: it not only exerts a direct effect of “technological empowerment reducing local expenditure” but also generates a spatial spillover effect of “resource agglomeration affecting peripheral expenditure.” These findings provide direct practical guidance for formulating differentiated digital healthcare development policies and optimizing the allocation of regional medical resources.

Overall, the results of the spatial heterogeneity analysis reveal that the digital economy exhibits a dual-effect mechanism of “expenditure reduction-siphoning” across different regions. In the future, efforts should be further made to strengthen the digital capacity building in the Central region, prevent it from being marginalized in the national digital economy development, and promote regional coordination and the equitable development of healthcare.

### 4.5 Mediating effect analysis

To further examine the mechanism through which the digital economy affects residents’ medical expenditure, this study employs a three-step stepwise regression approach to test the mediating effect, using the CPI as the mediating variable. The regression results are presented in Table 8.

**Table 8:** Mediation effect regression results.

Variable	(1)	(2)	(3)
	OOP	DEI	OOP
DEI	−12.234*** (−10.87)		−12.281*** (−10.89)
CPI		0.323** (0.57)	0.296 (0.90)
Control	Yes	Yes	Yes
obs	150	150	150
*R* ^2^	0.4439	0.0022	0.4470

*, **, and *** in Tables 6–10 denote significance at the 10%, 5%, and 1% levels, respectively.

The first-stage regression results indicate that the digital economy has a significant inhibitory effect on residents’ medical expenditure, with a coefficient of −12.23, significant at the 1% level. This represents the total effect of the digital economy on medical expenditure. In the second stage, the regression results show that the digital economy significantly promotes the rise of the CPI, with a coefficient of 0.323 and significance at the 5% level. It is important to clarify that the coefficient of 0.323 corresponds to the original data scale after standardization, indicating that for each unit increase in the digital economy index, the CPI increases by 0.323 percentage points. This positive relationship may stem from the cost-driving effects associated with digital transformation, such as infrastructure investments and technological upgrades, which are transmitted to consumer prices. It indirectly affected the supply and demand as well as the price level of goods and services (32, 33).

The third-stage results, which include both the digital economy and CPI in the regression, show that the direct effect of the digital economy on medical expenditure is −12.28, while the mediating effect through CPI is 0.096. The proportion of the mediating effect is calculated as (0.323 × 0.296) / |−12.23| ≈ 0.096/12.23 ≈ 0.0078, or 0.78%. This indicates a masking effect, where the digital economy’s tendency to increase CPI partially offsets its direct expenditure-reducing effect. However, since the direct effect remains dominant, the overall impact of the digital economy on medical expenditure remains significantly negative.

These findings reveal a complex mechanism: while the digital economy enhances medical service efficiency and reduces costs through technological empowerment (direct effect), it also generates upward pressure on consumer prices (mediated effect), thereby creating a masking effect that attenuates the overall expenditure reduction. This explains the stronger direct effect observed in the third stage compared to the total effect, highlighting the dual nature of the digital economy’s impact on residents’ medical expenditure.

### 4.6 Threshold effect analysis

To explore potential nonlinearities in the relationship between the digital economy and residents’ medical expenditure, this study employs a panel threshold regression model. The regional population ageing level (Population Ageing, Pa) is introduced as the threshold variable to test for segmented effects, hypothesizing that the impact of the digital economy may not be uniform across different stages of demographic aging. The model specification is as follows:


$$\begin{array}{c} \mathrm{OO}P_{\mathrm{it}}=\mu_{i}+\beta_{1}\mathrm{DE}I_{\mathrm{it}}I(q_{\mathrm{it}}\leq \gamma_{1})+\beta_{2}\mathrm{DE}I_{\mathrm{it}}I(\gamma_{1}<q_{\mathrm{it}}\leq \gamma_{2})\\ +\beta_{3}\mathrm{DE}I_{\mathrm{it}}I(q_{\mathrm{it}}>\gamma_{2})+\beta_{k}Z_{\mathrm{it}}+\varepsilon_{\mathrm{it}} \end{array}$$

**Figure 3:**
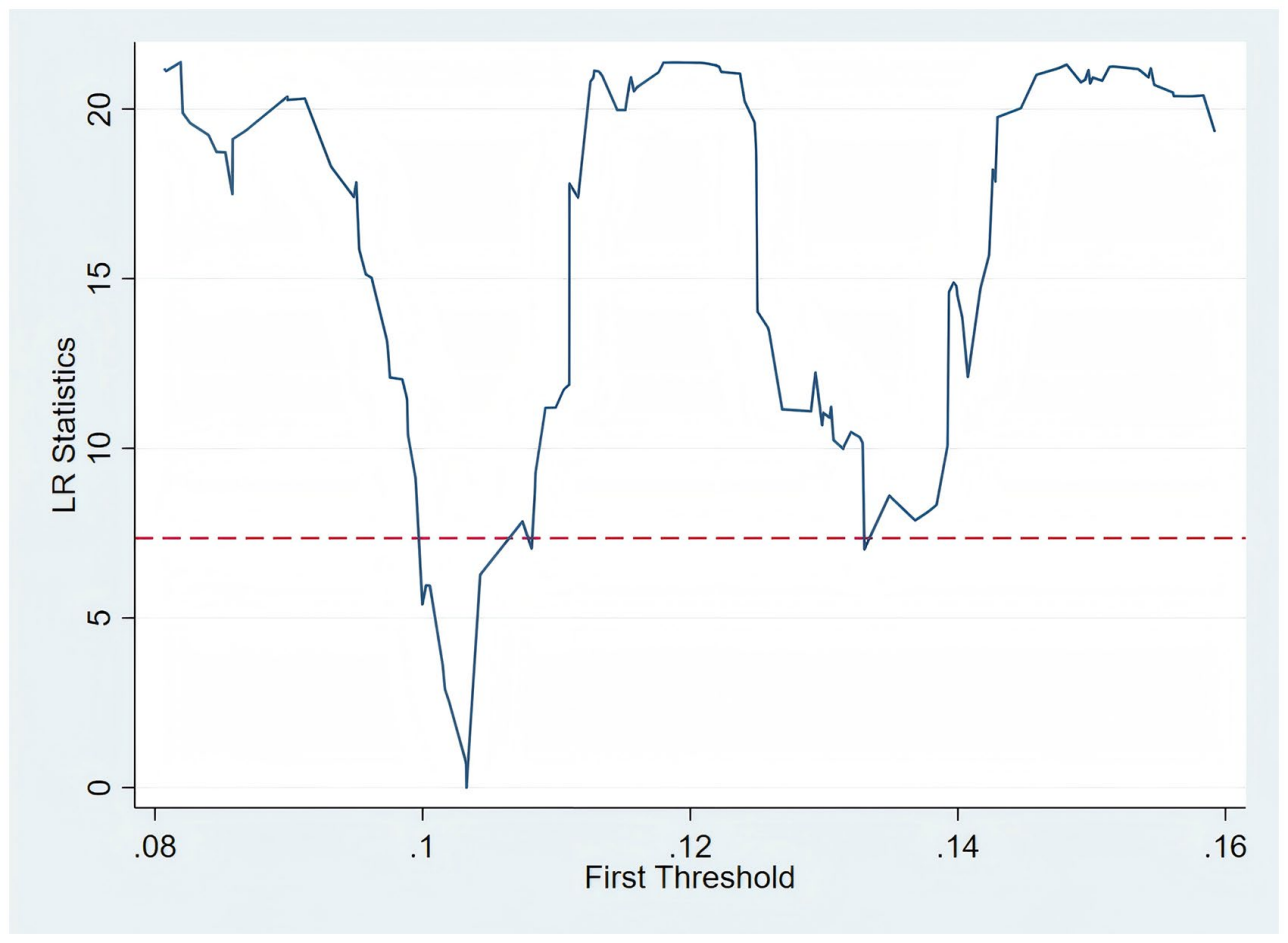
Likelihood ratio test result.

Here, $q_{\mathrm{it}}$ is the threshold variable (Pa), γ represents the threshold value(s) to be estimated, $\mu_{i}$ is the individual fixed effect, $\beta_{1}$ , $\beta_{2}$ , $\beta_{3}$ are the coefficients of the digital economy index (DEI) in different regimes, and $\varepsilon_{\mathrm{it}}$ is the error term. The likelihood ratio (LR) test for the existence of a threshold is presented in Figure 3. The test results, summarized in Table 9, indicate that the single-threshold model is statistically significant at the 5% level (F-statistic = 22.30, *p* = 0.032), while the double- and triple-threshold models fail to achieve statistical significance. This supports the adoption of a single-threshold specification.

**Table 9:** Threshold test results.

Threshold variable	Threshold number	F-statistics	*P*-value	Threshold value	95% confidence interval
Pa	single	22.30	0.032	0.1033	(−9.2792, −0.2215)
	double	11.90	0.152	0.1092	(−4.8383, 7.5114)
	Triple	4.10	0.840	0.1330	(−5.7962, 2.3384)

Based on the single-threshold model, the estimated threshold value for the population ageing level is 10.33%. The regression results in Table 10 suggest a structural change: when the ageing level is below this threshold, the coefficient for the digital economy is negative and significant ( $\beta_{1}$ = − 4.750, *p* < 0.05), indicating a significant inhibitory effect on medical expenditure. However, when the ageing level exceeds 10.33%, the coefficient becomes statistically insignificant ( $\beta_{2}$ = 1.337, *p* > 0.10).

**Table 10:** Threshold regression results.

variable	Pa
Pa < 0.1033	−4.750** (−2.08)
Pa > 0.1033	1.337 (0.43)
Obs	150
*R* ^2^	0.3598

*, **, and *** in Tables 6–10 denote significance at the 10%, 5%, and 1% levels, respectively.

While the LR test is significant, a crucial caveat must be highlighted. The 95% confidence interval for the estimated threshold, as shown in Table 9, is exceptionally wide, spanning from −9.28 to −0.22. This wide interval, which includes implausible negative values for a population ageing rate, casts considerable doubt on the precision and stability of the estimated threshold value of 10.33%. Therefore, the inference of a specific threshold at this exact value should be treated with extreme caution. A more conservative and statistically sound interpretation is that the relationship between the digital economy and medical expenditure is likely moderated by population ageing, but the exact point of structural change is not precisely identified by our model. The loss of significance in the high-ageing regime can be explained by several factors:

1. Changing demand structure: In highly aged populations, healthcare needs shift towards complex, continuous chronic disease management and long-term care, which are less amenable to the efficiency gains offered by current digital technologies focused on streamlining acute care and information access.
2. Low digital adoption among the older adults: The well-documented “digital divide” means the older adults has lower adoption rates of digital health tools. This fundamentally weakens the mechanism through which the digital economy could reduce costs, as the target user base is less engaged.
3. Offsetting effects: The potential cost-reducing effects of digitalization in some areas (e.g., administrative efficiency) may be offset by new costs associated with aging, such as the need for more expensive medical technologies or human-intensive care that digital tools cannot replace.

In conclusion, the analysis provides suggestive evidence of a diminishing marginal effect of the digital economy in the context of population aging. However, the imprecise threshold estimate necessitates further investigation. Future research should employ larger datasets, alternative model specifications, and perhaps different measures of aging or digital economy penetration to more robustly identify if and where a true threshold effect exists. Policy implications should therefore focus on the general moderating role of aging rather than designing interventions based on a specific, potentially unstable, critical value.

## 5 Discussion

### 5.1 Spatial effects of the digital economy on residents’ healthcare expenditure

Using panel data from 30 Chinese provinces between 2017 and 2021, this study first examines the effects of the digital economy on the medical expenditure of its citizens using a Spatial Durbin Model. This study performed multicollinearity analysis and modified the spatial weight matrix to guarantee the model’s correctness and robustness of the model and its outcomes. The model’s endogeneity problems were addressed by using the percentage of businesses that conducted e-commerce transactions compared to all businesses as an instrumental variable. In order to investigate the diverse impacts of various geographical locations on the model’s output, this study also used the Spatial Durbin Model. Furthermore, a three-stage regression mediation effect model and a threshold effect model were developed to investigate the processes by which the digital economy influences residents’ medical expenditure.

The results of the baseline model show that the digital economy has a primarily inhibitory effect on residents’ medical expenditure, which can be substantially ascribed to the technological advantages of the digital economy in areas like information management and transmission. The digital economy has streamlined the healthcare service process by creating online healthcare platforms, telemedicine services, and healthcare information systems. This has reduced medical visit-related income loss and saved patients money on transportation. In the meantime, the emergence of e-commerce platforms has increased transparency in the medicine procurement process, enabling patients to compare costs across various channels and select more reasonably priced goods. Drug providers have lowered their pricing due to competition in e-commerce platforms, which has further reduced consumers’ prescription costs.

The results of the regional heterogeneity analysis and the Spatial Durbin Model show that the digital economy has a significant spatial spillover effect on residents’ medical expenditure, exhibiting the characteristic of “local expenditure reduction and neighboring expenditure increase.” This “siphon effect” can be attributed to the concentration of high-quality healthcare resources, such as specialized doctors and advanced medical equipment, in digitally advanced regions. These regions leverage their technological and information advantages to create more attractive online medical platforms and service models. Consequently, this may draw both human capital and patient flows from neighboring, less digitally developed areas. Residents in neighboring regions, facing a relative decline in local healthcare service quality or availability, might be compelled to seek medical care across regions, thereby incurring higher travel, accommodation, and potentially higher medical costs, leading to increased expenditure.

The research of regional heterogeneity also shows that medical expenditure in both the eastern and western areas are somewhat inhibited by the digital economy. On the other hand, only government action has significantly reduced the medical expenditure on the people in the central region. This implies that the central region is caught in an “underdevelopment trap,” emphasizing the necessity of preventing the “Matthew effect” from escalating regional health disparities and avoiding the dangers of regional development imbalances.

### 5.2 Mechanisms of the digital economy’s impact on residents’ medical expenditure

To examine the mechanisms through which the digital economy affects residents’ medical expenditure, this study employed mediation and threshold effect models. The regression results in Table 8 indicate that the direct effect exceeds the total effect, suggesting the presence of additional pathways that attenuate the expenditure-reducing impact. Specifically, the CPI demonstrates a significant masking effect in the relationship. The identified masking effect reveals two competing channels, creating a dynamic antagonism rather than a simple linear relationship between the digital economy and medical expenditure.

The transmission mechanism of the CPI’s mediating effect operates through the following pathways: First, the deep integration of the digital economy with traditional industries requires substantial investments in infrastructure upgrades, system construction, and technical training (19). Enterprises typically pass these digital transformation costs to consumers through price increases for products and services, thereby driving the rise of the overall CPI level. Second, the digital economy reshapes consumption patterns, shifting preferences from basic functional consumption toward more personalized and high-quality consumption (20), which further elevates the general price level.

The essential characteristics of healthcare services amplify this masking effect. As a state-provided public benefit with low price elasticity, healthcare services exhibit essential nature and non-deferrable consumption attributes. Patients ultimately bear higher actual medical expenditures due to the combined effect of healthcare’s low price elasticity and the CPI increase driven by digital economy expansion. This conclusion aligns with findings from Li (30). This mechanism particularly exacerbates impacts on low-income regions and reduces 0.78% of the direct cost-saving benefits. Fundamentally, the masking effect reflects the contradiction between efficiency improvement and distribution imbalance within the digital economy, revealing the complexity of technology dividend transmission and distribution conflicts (33).

The threshold effect analysis further enriches our understanding. As shown in Table 10 and Figure 3, when population aging serves as the threshold variable, the impact of the digital economy on medical expenditure transitions from significant to insignificant. This indicates that population aging represents more than a demographic phenomenon—it substantially alters medical demand patterns, technology acceptance capacity, and healthcare system functioning. Consistent with the research of Feng, the cost-control mechanism of the digital economy thus loses its effectiveness in highly aged societies (34).

This transition occurs because the digital economy’s primary role in low-aging contexts involves replacing inefficient offline activities and enhancing efficiency. It effectively reduces personal medical expenditures by serving populations with high technological adoption rates and addressing relatively straightforward medical needs. However, as aging progresses, primary medical demands shift toward more complex, continuous, and human-intensive integrated care requirements. The significant digital gap among older adults, combined with the limited effectiveness of current digital technologies in replacing essential expensive services—sometimes even generating new costs—transforms the digital economy from a “substitute” into merely a “supplement” or even a “bystander.” Thus, aging’s structural pressures overwhelm the digital economy’s cost-reducing effects, rendering them statistically insignificant. Addressing medical expenditure challenges in super-aged societies requires more comprehensive approaches, including integrated care models, age-friendly technological innovations, long-term care insurance, and care workforce expansion, moving beyond reliance solely on healthcare informatization and digitization.

Furthermore, In the spatial econometric model, the essence of the time fixed effect is to control all common shocks that vary over time but not across individuals, with its core assumption being that the controlled factors exert a consistent impact on all research individuals at the same point in time. Nationwide uniformly implemented policies, such as the national requirement for the integration of urban and rural medical insurance in 2016, have a consistent impact on all provinces in the temporal dimension, meaning all provinces face the same policy requirements at the same time and such impact does not vary with the individual differences of provinces. This exactly matches the function of the time fixed effect, which is to absorb common time shocks. In contrast, it is difficult to directly eliminate the interference of policies promoted with regional differences, such as the regional disparities in the rollout pace of DRG/DIP—coastal regions taking the lead over central regions—and local medical payment reforms. In such cases, the policy shocks are not time effects common to all individuals, but rather the interaction effects between time and individuals. Therefore, it is impossible to exclude the interference of such differentiated policies. Hence, future research should supplement the discussion on the potential impacts of these uncontrolled policy factors, as well as quantitatively assess the extent to which the omission of these variables may interfere with the core estimation results.

The limitations of this study primarily manifest in following aspects. First, due to data constraints, the study is based on Chinese city data, future extensions to other countries or regions would be beneficial to verify the universality of the findings from this study. Second, the exact point of structural change is not precisely identified by our model, Future research should focus on identify if and where a true threshold effect exists.

## 6 Conclusions and recommendations

The following are the primary conclusions: First, the digital economy has a siphon effect on nearby regions even when it greatly reduces medical expenditure locally. Second, there are notable regional differences in how the digital economy affects the medical expenditure. In particular, all effects are negligible in the center region, while both direct and indirect effects are large in the eastern and western regions. Third, medical expenditure is largely influenced directly by the digital economy, but the CPI has a big masking impact on this link. Fourth, until aging reaches a certain point, the digital economy greatly reduces medical expenditure; after that, its influence is statistically negligible.

This study proposes the following recommendations:

First, enhance the construction of digital infrastructure and promote the in-depth integration of the digital economy into the medical field. Build a hierarchical and interconnected digital medical infrastructure network, and facilitate the cross-regional interconnection and sharing of medical data such as electronic medical records and examination results. Focus on strengthening the digital configuration of primary medical institutions; through fiscal subsidies and communication tariff incentives, improve the level of network coverage and equipment allocation in rural and remote areas, effectively narrow the urban–rural gap in digital medical care, and enable grassroots residents to conveniently access high-quality medical resources.

Second, promote coordinated regional development and narrow the digital gap in the central region. Establish a paired assistance mechanism for digital medical care between eastern developed regions and central cities, to facilitate the flow of high-quality factors such as technology and talents to the central region. Set up special industrial funds and attract digital health enterprises to settle in the central region through preferential tax policies, so as to boost the development momentum of the digital health industry in the central region.

Third, improve the price and policy coordination mechanism to ensure the effective transmission of technological dividends. Formulate a scientific pricing system for digital medical services, set reasonable prices for new services such as remote diagnosis and treatment and AI-assisted diagnosis, link the application of digital medical care with medical insurance payment, and guide medical institutions to take the initiative to upgrade services through policy incentives. Build a digital platform for centralized drug procurement, realize the whole-process supervision of drug circulation, reduce the costs of intermediate links, and ensure that the efficiency gains brought by technology are translated into tangible benefits for people’s livelihood.

Fourth, actively promote the aging-appropriate innovation of digital technology to address the challenges of aging. Promote the aging-appropriate transformation of health-related apps and online medical platforms, retain user-friendly functions such as voice interaction and large-font display, and develop proxy services for family members. Encourage the older adults to use intelligent health devices through consumption subsidies, set up assistance stations for older adults in community health service centers to provide on-site guidance for digital services. Improve the long-term care insurance system and the care talent training system, promote the integrated model of “intelligent monitoring + home care + community services,” and enhance the older adults sense of gain from digital health services.

## Data Availability

Publicly available datasets were analyzed in this study. This data can be found at: https://data.stats.gov.cn/easyquery.htm?cn=E0103; https://www.stats.gov.cn/sj/ndsj/; https://nsd.pku.edu.cn/docs/20221017131046905230.pdf.
